# Overcoming the Challenges to Clinical Development of X-Linked Retinitis Pigmentosa Therapies: Proceedings of an Expert Panel

**DOI:** 10.1167/tvst.12.6.5

**Published:** 2023-06-09

**Authors:** David G. Birch, Janet K. Cheetham, Stephen P. Daiger, Carel Hoyng, Christine Kay, Ian M. MacDonald, Mark E. Pennesi, Lori S. Sullivan

**Affiliations:** 1 Retina Foundation of the Southwest, Dallas, TX, USA; 2 Foundation Fighting Blindness, Columbia, MD, USA; 3 Human Genetics Center, School of Public Health, University of Texas Health Science Center, Houston, TX, USA; 4 Radboud University, Nijmegen, The Netherlands; 5 Vitreoretinal Associates, Gainesville, FL, USA; 6 University of Alberta, Edmonton, Canada; 7 Casey Eye Institute, Oregon Health & Science University, Portland, OR, USA

**Keywords:** X-linked retinitis pigmentosa (XLRP), clinical trials, genotype/phenotype relationships, natural history, outcome measures

## Abstract

X-linked retinitis pigmentosa (XLRP) is a rare inherited retinal disease manifesting as impaired night vision and peripheral vision loss that progresses to legal blindness. Although several trials of ocular gene therapy for XLRP have been conducted or are in progress, there is currently no approved treatment. In July 2022, the Foundation Fighting Blindness convened an expert panel to examine relevant research and make recommendations for overcoming the challenges and capitalizing on the opportunities in conducting clinical trials of *RPGR*-targeted therapy for XLRP. Data presented concerned *RPGR* structure and mutation types known to cause XLRP, *RPGR* mutation–associated retinal phenotype diversity, patterns in genotype/phenotype relationships, disease onset and progression from natural history studies, and the various functional and structural tests used to monitor disease progression. Panel recommendations include considerations, such as genetic screening and other factors that can impact clinical trial inclusion criteria, the influence of age on defining and stratifying participant cohorts, the importance of conducting natural history studies early in clinical development programs, and the merits and drawbacks of available tests for measuring treatment outcomes. We recognize the need to work with regulators to adopt clinically meaningful end points that would best determine the efficacy of a trial. Given the promise of *RPGR*-targeted gene therapy for XLRP and the difficulties encountered in phase III clinical trials to date, we hope these recommendations will help speed progress to finding a cure.

**Translational Relevance:**

Examination of relevant data and recommendations for the successful clinical development of gene therapies for *RPGR*-associated XLRP.

## Introduction

X-linked retinitis pigmentosa (XLRP) is a severe, aggressive, inherited retinal disease characterized by progressive photoreceptor deterioration and loss eventually leading to blindness.^1^^,^^2^ Pathogenic variants associated with XLRP affect predominately male individuals. Female carriers of a disease-causing variant sometimes can be affected clinically, and, in these cases, typically present with a milder phenotype than male patients, potentially due in part to random or skewed X chromosome inactivation.^1^^–^^5^ The most common causes of XLRP are pathogenic variants in two genes, retinitis pigmentosa guanosine triphosphatase regulator (*RPGR*) and *RP2*, accounting for approximately 70% and 20% of cases, respectively.^6^^–^^11^ Currently, there is no treatment for XLRP. Although several investigational XLRP therapies targeting the *RPGR* gene have met with early successes in phase I/II clinical studies,^12^^–^^14^ the only phase II/III study to read out to date, the XIRIUS study of adeno-associated virus serotype 8 (AAV8) vector-based gene therapy, cotoretigene toliparvovec, failed to meet its primary end point.^15^

On July 23, 2022, The Foundation Fighting Blindness convened a virtual meeting of experts in ophthalmology and genetics to discuss the challenges and opportunities in the clinical development of XLRP genetic therapies. Key topics were:

• Delineating the target patient population, including defining pathogenic RPGR genetic mutations and associated retinal disease clinical phenotypes
• Establishing XLRP disease course and identifying factors associated with disease outcomes
• Selecting the structural and functional tests best suited to measure outcomes

The Expert Panel's goal was to formulate recommendations for designing XLRP gene therapy clinical studies to provide the best chance of successful treatment development. This article summarizes the meeting's presentations and discussions and the panel's recommendations.

### *RPGR* Mutations and Retinal Phenotypes

Located on Xp11.4,^16^^–^^18^ *RPGR* was first identified in 1996 as the gene responsible for the most common form of XLRP.^19^^,^^20^ The gene has a complex expression pattern, with >20 spliced isoforms. *RPGR* is found in a variety of tissues throughout the body, including in the eyes, brain, lungs, testes, and kidneys.^16^^,^^17^^,^^20^^,^^21^ The 2 most common variants are a “normal” isoform, which contains 19 exons encoding the full-length protein, and a retina-specific isoform that contains exons 1 to 14 and ends with an extended open reading frame (ORF) at the exon 15 position. This ORF15 isoform represents the predominant isoform in the retina, and mutations in this isoform, particularly in the ORF15 mutational “hot spot,” are known to be responsible for many XLRP cases.^7^^,^^21^^,^^22^ ORF15 encodes >500 amino acids and contains polymorphic repetitive elements. The most repetitive sequence comprises 27 imperfect direct repeats, each of which is 15 to 33 nucleotides long.^7^ Polymorphic in-frame insertions and deletions are extremely common throughout this repetitive sequence. These qualities make sequencing ORF15 challenging and explain why reliable sequence data can be difficult to obtain.^23^ This is particularly true for female individuals, because the second X chromosome typically has a different polymorphic sequence in this region, which complicates alignment. There are several commercial laboratories that offer test panels, including ORF15, that have optimized their sequencing protocols to enable analysis of this difficult region. Even with these enhancements, testing is ideally first performed on affected male patients. To date, >700 unique pathogenic *RPGR* mutations have been associated with XLRP and represent a wide variety of types, including missense, nonsense, frameshift, splice, and insertion/deletion.^24^^–^^26^ Over 300 mutations in exons 1 to 14 and nearly 350 mutations in ORF15 have been identified as causing an X-linked retinal disease.^25^ Although recurrent mutations in *RPGR* occur, they are not common and often share a common ancestor rather than arising independently.^5^^,^^23^

Limits on data accessibility, as well as lack of information on phenotypic and functional effects associated with mutations, hamper the clinical use of genetic variant information. The information on *RPGR* variants, as with other genes, is spread across multiple individual databases, some of which are proprietary.^24^^–^^27^ To address these obstacles, the US National Human Genome Research Institute has created the Clinical Genome (ClinGen) program with the goals of sharing genomic and phenotypic information through centralized and federated databases and developing standards to support the clinical annotation and interpretation of mutations. In doing so, there will be a set of validated pathogenic mutations.^27^ This endeavor is relevant to developing *RPGR*-targeted therapy for XLRP in part because discussions with the US Food and Drug Administration (FDA) have suggested that a genetic therapy must target a gene with variants that have been validated for pathogenicity (S.P. Daiger, personal communication). The FDA has recognized ClinGen as a valid source of human genetic mutation interpretation data, and work on the X-linked retinal disease domain is on-going.^27^^,^^28^

Clinical phenotypes associated with pathogenic *RPGR* mutations fall within the broad category of progressive diffuse photoreceptor diseases. Traditionally, they have been classified into the subgroups “rod dystrophy,” rod-cone dystrophy (typical retinitis pigmentosa [RP]), cone-rod dystrophy (CRD), and cone dystrophy (CD) based on the pattern and extent of rod versus cone photoreceptor degeneration from full-field flash electroretinography (ffERG).^29^^,^^30^ The first two categories represent a somewhat artificial delineation, because RP, even if it arises from mutations that primarily affect rod photoreceptors, will eventually involve cone function loss and cone degeneration due, for example, to a reduction in rod-derived cone viability factor as rods die.^31^ In research, rod-cone dystrophy is either referred to as a form of RP or vice versa, or the terms are used synonymously.^29^^,^^32^^,^^33^ Clinically, RP is preferred and will be used here.

In the context of defining target populations for *RPGR*-targeted gene therapy trials, it is necessary to differentiate between rod- and cone-predominant dystrophy phenotypes due to their differing clinical appearance and progression patterns. RP, which includes simple/nonsyndromic, syndromic, and systemic disease, is the most common retinal dystrophy, with a worldwide prevalence ranging from approximately 1:3700 to 1:8300, reflecting regional differences.^1^^,^^2^^,^^30^^,^^34^ Of the different RP forms, XLRP is the most severe and represents about 10% to 20% of RP cases.^1^^,^^2^^,^^6^^,^^35^ Nyctalopia and peripheral visual field loss are early hallmark RP manifestations, followed by central visual acuity decline later in the disease course. Retinal examination findings include retinal pigment epithelium atrophy, “bone spicule” pigmentary deposits, and retinal vessel attenuation in the fundus; posterior subcapsular cataracts; and dust-like particles in the vitreous.^29^^,^^30^^,^^32^ Diminished or absent response to dark-adapted dim flash and delayed and diminished or absent a- and b-wave dark-adapted response to a bright flash are characteristic changes in electroretinogram (ERG) patterns.^29^^,^^32^^,^^36^

The cone dystrophies—CRD, in which cone dysfunction precedes rod dysfunction, and CD, in which rod photoreceptors remain unaffected—are less common than RP, with a global prevalence of approximately 1:30,000 to 1:40,000.^37^^,^^38^ Although an X-linked inheritance accounts for only about 1% of CRD cases, *RPGR* mutations are the cause in the majority of them.^22^^,^^39^^,^^40^^,^^41^ Early symptoms include decreased visual acuity, photophobia, and color vision disturbances. Central scotoma is common. With disease progression, individuals with CRD will develop patchy loss of peripheral vision and nyctalopia as rods degenerate. Retinal examination may reveal a bullseye maculopathy, but the macula may appear normal in early stages. Individuals with CRD eventually develop the bone spicule pigmentary deposits and retinal vessel attenuation seen in RP.^37^^–^^39^ Early characteristic ffERG patterns for CD are delayed, reduced, or absent light-adapted flash and 30 Hz flicker responses with normal rod ERG responses. However, later in the disease, rod responses can be diminished.^29^^,^^38^^,^^42^

Age of onset for X-linked CD and CRD arising from *RPGR* mutations is comparatively later than that for *RPGR*-associated XLRP. Male patients with XLRP typically are diagnosed in childhood or early adolescence, whereas X-linked CD and CRD symptom onset typically occurs in adulthood.^40^^,^^43^^,^^44^ A retrospective, multicenter study involving 74 male individuals with *RPGR*-associated retinal dystrophies in the Netherlands by Talib et al. found the median age of onset for those with XLRP (*n* = 52) was 5 years compared with 23 years for those with CD/CRD (*n* = 22).^43^ This study also illustrated the different disease progression patterns among these phenotypes. Based on best corrected visual acuity (BCVA), about 20% of individuals with XLRP, compared with about 55% of individuals with CD or CRD, were blind 40 years of age.^43^

Most *RPGR* mutations (approximately 70%) present as the XLRP phenotype, with a much smaller proportion leading to X-linked CRD (up to 23%) and an even smaller proportion resulting in CD (approximately 7%).^10^^,^^22^^,^^43^ Data from studies of families with X-linked CRD and/or CD have shown that mutations associated with these conditions predominantly occur in ORF15 and are clustered at the 3′ end.^22^^,^^39^^,^^40^^,^^45^^,^^46^ For example, a genetic analysis of X-linked CD among 2 families of Northern European decent by Yang et al. linked the associated mutations to chromosomal locus *COD1*, which maps to a region harboring *RPGR*, and identified 2 mutations in *RPGR* ORF15 (ORF+1343_1344delGG and ORF+694_708del15) that were present in affected individuals but not in control subjects.^45^ Thiadens et al. identified 2 frameshift mutations in the *RPGR* ORF15 3′ region that resulted in premature protein truncation in an analysis of 2 families from the Netherlands with X-linked CD.^40^ In contrast, evidence suggests *RPGR* mutations resulting in the XLRP phenotype may be concentrated in exons 1 to 14 and the proximal part ORF15, with a “watershed” zone of mutations in ORF15 occurring at about region 949 to 1047 that may be associated with either XLRP or X-linked cone dystrophies.^22^

### XLRP Natural History Related to *RPGR* Genotype

RPGR-related forms are among the most severe forms of RP. As noted above, XLRP symptom onset typically occurs in childhood or early adolescence.^43^^,^^44^^,^^47^ However, these data largely come from retrospective cohorts in which individuals self-reported symptoms.^43^^,^^47^ Because it can be difficult for children to discern vision decline, estimates from these types of studies may not accurately represent the age of onset for retinal pathological changes. There is evidence to suggest that rod function loss in XLRP can begin shortly after birth.^48^ A study by Birch et al. among 14 male infants and children at risk of developing XLRP used steady-state full-field pupillometry as a function of retinal illuminance to detect early rod function loss. Participants were determined to be at risk of XLRP if they had ≥2 male relatives known to have early-onset night blindness and visual impairment symptoms, an absence of male-to-male symptom transmission in the families, and only partially affected female relatives. All mothers were XLRP carriers. Mean age of initial pupillometric test was 3.63 years (range = 4 months to 8 years). After age 5 years, 9 participants showed reduced ERGs and were diagnosed with XLRP. Seven of these participants had significantly elevated pupil thresholds on the initial test (*P* < 0.05), and all had elevated thresholds on subsequent annual visits. The remaining 5 participants with normal pupil thresholds also subsequently had normal ffERGs after age 5 years and exhibited no XLRP symptoms.^48^ These results indicate XLRP can be detected at a very young age among individuals determined to be at risk through pedigree analysis and carrier identification.

Several studies have investigated disease severity and progression rate in individuals with *RPGR*-associated XLRP based on genotype and other factors.^43^^,^^47^^,^^49^ A retrospective study by Di Iorio et al. investigated age of onset and disease severity in 48 individuals with XLRP from 31 families seen at a single center in Naples, Italy. Although mean self-reported age of onset did not differ significantly (7.6 years versus 6.3 years, respectively), individuals with mutations located in ORF15 exhibited faster disease progression as measured by BCVA than those with mutations in exons 1 to 14: 0.044 logMAR/year versus 0.011 logMAR/year, respectively (*P* < 0.001).^47^ In the study by Talib et al. in the Netherlands, which included individuals with *RPGR*-associated RP, CD, or CRD, BCVA also declined more quickly for individuals with mutations in ORF15 (0.022 logMAR/year) compared with those with mutations in exons 1 to 14 (0.015 logMAR/year). However, the difference was not statistically significant.^43^ Similar to Talib et al., Di Iorio found that 50% of individuals with XLRP had low vision based on BCVA at age 48 years.^47^ However, although a similar proportion had legal blindness based on BCVA at age 40 years in both studies (approximately 20%), the study by Di Iorio et al. found a step decline in vision loss occurred in the fourth decade of life such that 50% of individuals had reached this threshold by age 51 years. In addition, Di Iorio et al. showed that legal blindness based on Goldmann visual field (GVF) testing (versus BCVA) occurred much earlier, with 50% reaching this threshold at age 26 years.^47^ Finally, Fahim et al. investigated how genetic factors, including allelic diversity and modifier genes, impact XLRP phenotype in a retrospective analysis of US registry data. The cohort comprised 98 male subjects from 56 families with 44 different *RPGR* mutations. In addition, the investigators genotyped individuals for coding single nucleotide polymorphisms (SNPs) in 4 modifier genes with products that interact with RPGR protein: *RPGRIP1*, *RPGRIP1L*, *CEP290*, and *IQCB1*. Individuals were stratified based on ffERG and Humphrey visual field (VF) testing into clinical phenotypes of grade 1 (mild), 2 (moderate), or 3 (severe). The findings showed a range of disease severity between and within families.^49^ In contrast to the study by Di Iorio et al.,^47^ mutations in *RPGR* exons 1 to 14 resulted in a more severe phenotype than those in ORF15: individuals with mutations in this area were more likely to have moderate or severe disease (*P* = 0.016), whereas the disease severity distribution for individuals with mutations in ORF15 was roughly equal across all 3 phenotype grades. This study also found that individuals with null alleles were more likely to have moderate or severe disease (*P* = 0.0038), whereas the disease severity distribution for individuals with mutations resulting in variant protein was roughly equal across all three phenotype grades. Two coding SNPs in *RPGRIP1L* (R744Q) and *IQCB1* (I393N) interacting with *RPGR* also were significantly associated with more severe disease (*P* < 0.05).^49^ Taken together, these findings illustrate the complexities of determining genotype/phenotype relationships for XLRP and the potential influence of mutations in genes beyond *RPGR*.

### Meaningful Functional and Structural Tests in XLRP

XLRP is associated with progressive visual function loss revealed by testing visual acuity and VF. However, because visual acuity can be preserved until later stages of RP progression,^32^ VF testing may be more useful to track visual function in XLRP and may be a better marker of disease progression. In the studies by Talib et al. and Di Iorio et al., for example, individuals with XLRP experienced annual declines in BCVA of 0.011 logMAR to 0.044 logMAR, and about 20% of individuals had legal blindness based on BCVA at age 40 years.^43^^,^^47^ However, Di Iorio et al. showed that legal blindness based on GVF testing occurred much earlier.^47^ Similarly, a long-term (up to 29 years), retrospective analysis conducted by Xu et al. of 275 GVF tests conducted in 52 individuals with RP of different etiologies found that the mean age of survival (Kaplan-Meier analysis) for legal blindness based on visual acuity was 11 years older than that based on GVF (*P* = 0.05). This study also illustrated the utility of GVF area in determining RP disease progression. For the entire cohort, annual GVF area loss rates varied significantly based on target size, with a faster deterioration rate seen for the smaller targets III4e (10.7%) and I4e (12.5%) than for the larger V4e target (7.5%; *P* < 0.001).^50^

Static automated perimetry's (SAP's) usefulness to evaluate VF in XLRP may vary based on the instrument used. Several studies have found that Humphrey static perimetry test-retest variability in RP can be high, especially in regions with low sensitivity of 8 to 12 dB.^51^^–^^53^ However, a recent study of Octopus 900 static perimetry in a small cohort of (*n* = 10) individuals with *RPGR*-associated RP showed good repeatability.^54^ In this study, average mean sensitivity was similar for the right and left eyes (7 dB and 6.8 dB, respectively), and the coefficient of repeatability for mean sensitivity was <2 dB.^54^ A larger study using Octopus 900 static perimetry with a customized 185-point radial grid, along with other metrics, to assess VF in 47 individuals with *RPGR*-associated retinopathy also generally showed good interocular symmetry, with variations between individuals and based on the measurement used. In this study, based on Octopus mean sensitivity values, the median annual rate of VF decline was 7.62%, and the overall annual rate of exponential decline was 4.67%.^55^

Fundus-driven perimetry, or microperimetry, is a solution for quantifying the central VF and monitoring progression in individuals with inherited retinal disease (IRD) who have relatively unstable fixation, offering test-retest repeatability and correlation to retinal pathology.^56^^,^^57^ Anikina et al. used mesopic microperimetry (Nidek MP-1; Nidek Co., Ltd.) with a radial 44-point test grid and a Goldmann size III target to characterize retinal function in a prospective case series in 76 children and adults (age range = 6.9–55.8 years) with *RPGR*-associated retinopathy. Mean follow-up was 2.8 years, and most participants had XLRP. The method resulted in a high test-retest reliability and good overall interocular symmetry in mean sensitivity (MS) and volumetric indices. Mean annual progression rates of MS, total volume, and central 3-degree field volume (0.82 dB/year, 0.04 dB-sr/year, and 0.01 dB-sr/year, respectively) were comparable with rates recorded in previous studies of full-field static perimetry measured with the Octopus 900 perimeter.^57^ Fundus-driven perimetry has recently utilized the Macular Integrity Assessment (MAIA) microperimeter, which has been an outcome measure in XLRP gene therapy clinical trials.^12^^,^^15^ In the first-in-human XLRP gene therapy phase I/II clinical trial, for example, one individual treated with codon-optimized AAV2 serotype 8 vector (AAV8.co*RPGR*) showed retinal sensitivity improvement on MAIA microperimetry in the treated eye from 0.5 dB to 6.6 dB after 3 months.^12^ In contrast, in the phase II/III XIRIUS study, treatment with cotoretigene toliparvovec had no significant impact on the proportion of eyes with ≥7 dB improvement at ≥5 of the 16 central loci of the 10-2 grid as assessed MAIA microperimetry after 1 year.^15^ The ≥7 dB improvement at ≥5 locations suggested as a positive primary outcome by regulatory authorities may be difficult to achieve in XLRP. A delta of ≥7 dB between treated and untreated eyes may be more achievable with, for example, the treated eyes gaining 3.5 dB, whereas untreated eyes lose 3.5 dB. Alternately, a significant slowing of the slope of progression may be a more realistic goal.

Several studies have investigated visual function loss in RP and XLRP using ffERG. Birch et al. showed that, over a 4-year interval in a cohort of 67 individuals with RP and measurable rod ERG at baseline, 64% and 60%, respectively, showed rod and cone ERG amplitude declines, with a larger annual change in rod ERG threshold versus cone ERG threshold.^58^ Individuals with XLRP (*n* = 10) exhibited a significantly higher annual rate of rod ERG threshold elevation (0.22 log/year) than those with autosomal dominant RP (0.1 log/year; *P* < 0.05).^58^ A separate study in 24 individuals with XLRP age 5 to 38 years and 100 control participants age 5 to 75 years used cone and rod ERG a-wave data obtained using high-intensity stimuli in the dark to determine visual function loss.^59^ Over a 4-year period, annual testing showed that rod response maximum amplitude declined by 45% in individuals with XLRP. Test-retest variability for a-wave maximum cone and rod responses was improved over that in b-wave peak-to-peak amplitude, indicating this measure could be useful to track photoreceptor function in XLRP natural history and treatment studies.^59^ Because most individuals with XLRP will not have a measurable ERG response by young adulthood, however, the test's utility is limited to younger cohorts. For example, in the study by Di Iorio et al., ERG responses were not detectable in 50% of the 48 individuals with XLRP, and those exhibiting a rod-cone pattern on ERG were significantly younger (mean age = 24.8 years) than those with undetectable ERG (mean age = 34.4 years; *P* = 0.027).^47^ In the study by Birch et al., which required participants to have measurable ERG at baseline, individuals with XLRP were younger (mean age = 14.7 years) than those with other types of RP (mean age = 27.7–34.8 years).^58^ Techniques are available for following sub-microvolt responses in more advanced patients, but these are difficult to standardize in a multicenter trial.^60^

Full-field stimulus testing (FST) has been used in natural history studies and clinical trials of gene therapy for rare IRDs, such as Usher syndrome type 2A and Leber congenital amaurosis.^61^^,^^62^ Developed to measure visual thresholds in individuals with severe vision impairment who were unable to fixate, the test holds promise for applications in XLRP, but has not been studied extensively in the condition.^61^

Structural tests also can be used to determine XLRP disease progression and may have advantages over VF as clinical trial outcomes measures because of the shorter duration needed to document change.^63^^,^^64^ In a study using spectral-domain optical coherence tomography (SD-OCT) to measure outer segment (OS) changes, 27 of 28 participants with XLRP had significant ellipsoid zone (EZ) width decreases after only 2 years.^63^ On average, EZ width decreased by 7.0% to 7.3% each year (*P* < 0.001), which is similar to the annual GVF decrease observed over 4 years by Xu et al. (7.2%).^50^ Test-retest measures had good agreement regardless of EZ width among individuals in the study with recessive or simplex RP (*n* = 20) who underwent SD-OCT twice on the same day.^63^ A subsequent study by Tee et al. that used SD-OCT to document disease natural history in 38 individuals with *RPGR*-associated RP found a positive correlation between EZ dimension and disease progression, with an overall annual exponential EZ width decline rate of 8.2%.^64^ However, EZ width is a linear measure and only samples a small portion of the region with preserved photoreceptors. Several small studies in RP have used *en face* OCT imaging to quantify the entire EZ area as proof-of-concept to support this measure's utility to monitor disease progression.^64^^–^^66^ For example, Hariri et al. took SD-OCT images from 24 individuals with autosomal dominant RP, manually inspected them to ensure the full extent of the EZ was within the scanning window, and used the manufacturer's software (Heidelberg Eye Explorer, version 1.9.10.0; Heidelberg Engineering) to generate 3-dimensional views.^65^ These views were subsequently used to create en face slab images from which two independent graders measured the EZ areas. The study found that there was good agreement between the graders and, using the mean results from the measurements, that EZ area decreased 13% from 2.67 mm^2^ to 2.40 mm^2^ over 1 year.^65^ In the study by Tee et al., which also generated EZ area en face images using high-density macular volume scans, the annual decline was 15.5%.^64^ Recently, progress also has been made in refining automated EZ area analysis using deep learning algorithms and in developing 3-dimensional metrics of the photoreceptor OS, with potential applications to assess disease progression and structural/functional relationships in RP.^67^ Relationships documented between OCT parameters and visual field parameters make the EZ area an appealing surrogate end point.^68^^,^^69^

### Considerations for Designing XLRP *RPGR* Gene Therapy Clinical Trials

The Expert Panel's recommendations for designing *RPGR*-targeted gene therapy clinical studies in XLRP are summarized below. These recommendations are based on careful consideration of the available literature, as well as the panel's experience in XLRP and *RPGR* research, including clinical trials. The panel hopes these recommendations can help guide discussions among investigators, industry, and regulators working toward the ultimate goal of curing affected individuals and preventing blindness.

#### Patient Selection and Stratification

 

• Expert genetic review boards for clinical trials are essential to help determine inclusion and exclusion criteria based on genetic mutations. For example, a board may make a recommendation that only pathogenic variants in RPGR be considered initially for inclusion in a clinical trial. Pathogenic variants in genes other than *RPGR*, might impact phenotype and confound analysis.
• At minimum, it is important to confirm that the mode of inheritance is X-linked, exclude an *RP2* mutation pathology, identify and document the pathogenic *RPGR* mutation, and define the break points and DNA level change of structural variants.
  ○ A pathogenic CHM mutation associated with X-linked choroideremia, should be excluded.
• Because cone-rod versus rod-cone phenotypes typically require different measures of progression, they should not be mixed in one arm of a trial.
• It is too early in our understanding of genotype/phenotype relationships and of modifier gene effects in XLRP to use specific mutations or mutation types to balance treatment groups or stratify trial participants.
  ○ However, trials should collect as much information as possible about the pathogenic mutations as part of standard genetic work-up in anticipation that technology will be available in the near future to allow investigators to better correlate XLRP genotype and phenotype.
• Because of the wide phenotypic variation among female carriers, clinical trials should include (or should focus on) only male participants and/or have separate trials or cohorts for affected female individuals.
• Given the rapid progression of vision loss, age <35 years is a reasonable inclusion parameter for an XLRP RPGR gene therapy trial because:
  ○ Participants must have residual microperimetry or static perimetry responses or residual EZ on OCT to allow for an outcome to be measured.
  ○ Children, adolescents, or young adults with XLRP may be the most likely age group to benefit.
• Because of the relationship between age and severity, it may be desirable to stratify by age; however, stratification beyond age 35 years is probably not meaningful.
• Gathering preliminary efficacy data from phase I/II clinical trials, although not their primary purpose, may help inform inclusion criteria, population stratification factors, and outcomes measures for phase III trials, allowing investigators to enrich later trials with the types of participants in which there was the largest efficacy signal.
  ○ Consideration should be given to increasing the number of patients in early clinical studies, because the volume of phase I/II data needed for this type of analysis is more than is typically collected in a clinical development program.

#### Trial Design, Outcome Measures, and End Points

• Although a 2-year phase III clinical trial may be sufficient, a longer duration (3–4 years) may be required to demonstrate treatment efficacy for an RPGR-targeted therapy for XLRP, particularly when using VF as an outcome measure.
• At present, the FDA guidance for industry for retinal disorder gene therapies recommends phase III trials include greater than or equal to two treatment arms of different doses in an effort to reduce potential bias and to add value as a dose-ranging control.^70^ Although reducing potential bias is very important in clinical trials, requiring that two dose levels of an investigational product be used in a gene therapy phase III trial is problematic because:
  ○ Having more than one therapy dose arm in a phase III trial dilutes the efficacy, safety, and tolerability outcomes data.
  ○ Determining the maximal tolerable dose is done early in clinical development (phase IB/II before a phase III trial. Thus, investigators may be concerned about the effectiveness of the lower of two doses given to patients in phase III.
• In general, when choosing tests for clinical trials, it is important to consider the participants’ tolerance thresholds for prolonged and repeated testing.
• Because visual acuity can be preserved until later in progression,^32^ this test is of limited use as an XLRP gene therapy clinical trial outcome measure, when the aim is to intervene earlier in the disease course. Regardless, this will be required by the regulators as a safety measure.
• As an outcome measure, ffERG is only relevant for pediatric or adolescent clinical trials, and multifocal ERG should not be used due to low signal-to-noise ratio in XLRP.
• We need to explore and fund research into using OCT findings, such as EZ changes and photoreceptor OS volume, to measure outcomes in XLRP gene therapy clinical trials, which could allow for shorter trial durations. Further identifying correlations to visual function will strengthen structural outcomes for regulators.
• Demonstrating a ≥7-dB change at ≥5 prespecified points on microperimetry is the standard primary end point for phase III trials based on FDA guidance,^71^ but is based on diseases such as glaucoma and may not be appropriate for *RPGR*-associated XLRP gene therapy.^15^ Rather, a small change in the slope of change can compound over years.
  ○ Thus, an alternative may be a slope analysis showing a progressive separation between treatment and control groups over time.
• Demonstrating improvement on treatment in function (e.g. microperimetry or static perimetry) and changes in structure (e.g. OCT) could be a useful compound end point in phase III trials because of its potential to show the benefit of therapy even if the primary end point is not met.

#### Conducting XLRP Natural History Studies

• Natural history studies are a critical component of an *RPGR*-targeted gene therapy clinical development program for XLRP, because the data they provide can help:
  ○ Determine cohort size needed to demonstrate efficacy.
  ○ Validate age as a disease progression biomarker for stratification.
  ○ Determine additional biomarkers that indicate fast versus slow progression, which could be used to select and stratify participants.
  ○ Determine the VF cutoff point for inclusion, which must allow for treatment efficacy measurement.
  ○ Validate efficacy end points selection, such as EZ changes or changes on microperimetry or static perimetry.
  ○ Determine expected disease progression over a certain amount of time to provide a benchmark against which to demonstrate lack of disease progression with treatment, which may be a reasonable measure of efficacy in XLRP (versus the higher bar of vision improvement).
• Ideally, natural history studies for XLRP should be conducted prior to conducting clinical trials and, given the typical small cohort size for a rare disease like XLRP, should be of 2 to 3 years’ duration.
  ○ However, because of the rapid disease progression with XLRP, a shorter natural history study of <2 years could be of value if the cohort size was large (approximately 200 participants) and the outcome measures focused on changes that could be detected in this time frame, such as EZ changes.
• It would be useful for the field if such data is made accessible in an open clinical trial data repository (there are a few) or via a publication.
